# Acute Effects of Cocoa Flavanols on Blood Pressure and Peripheral Vascular Reactivity in Type 2 Diabetes Mellitus and Essential Hypertension

**DOI:** 10.3390/nu14132692

**Published:** 2022-06-28

**Authors:** Anouk Tanghe, Elsa Heyman, Elodie Lespagnol, Jan Stautemas, Bert Celie, Jos Op ‘t Roodt, Ernst Rietzschel, Danusa Dias Soares, Nina Hermans, Emmy Tuenter, Samyah Shadid, Patrick Calders

**Affiliations:** 1 Department of Rehabilitation Sciences, Ghent University, 9000 Ghent, Belgium; anouk.tanghe@ugent.be (A.T.); jan.stautemas@ugent.be (J.S.); bert.celie@ugent.be (B.C.); 2 Univ. Lille, Univ. Artois, Univ. Littoral Côte d’Opale, ULR 7369-URePSSS-Unité de Recherche Pluridisciplinaire Sport Santé Société, F-59000 Lille, France; elsa.heyman@univ-lille.fr (E.H.); elodie.lespagnol@univ-lille.fr (E.L.); 3 Institut Universitaire de France (IUF); 4 School of Cardiovascular Diseases (CARIM), Maastricht University Medical Centre, 6229 HX Maastricht, The Netherlands; jos.op.t.roodt@telenet.be; 5 Department of Cardiology, Department of Internal Medicine, Ghent University Hospital, 9000 Ghent, Belgium; ernst.rietzschel@ugent.be; 6 Department of Physical Education, The Federal University of Minas Gerais, Belo Horizonte 6627, Brazil; danusa@eeffto.ufmg.br; 7 Department of Pharmaceutical Sciences, University of Antwerp, 2610 Antwerp, Belgium; nina.hermans@uantwerpen.be (N.H.); emmy.tuenter@uantwerpen.be (E.T.); 8 Department of Endocrinology, Ghent University Hospital, 9000 Ghent, Belgium; s.shadid@sro.ch

**Keywords:** type 2 diabetes, cocoa flavanols, vascular reactivity, blood pressure, antihypertensive drugs

## Abstract

Background: Type 2 diabetes mellitus (T2DM) is associated with a high risk of vascular complications. Interestingly, cocoa flavanols (CF) can exert beneficial vascular effects in non-diabetic subjects. However, these effects have only been scarcely studied in T2DM. Therefore, we performed a study to assess the effects on vascular reactivity of a single dose of CF (790 mg) in T2DM and whether certain antihypertensive drugs may modulate these effects. Methods: 24 non-diabetic and 11 T2DM subjects were studied in a cross-over design. Fasting blood samples, blood pressure (BP), and arterial vasoreactivity (flow-mediated dilation) were assessed before and 70 min after capsule ingestion. Muscle microvascular reactivity was only assessed after capsule ingestion. Age, waist-to-hip ratio, BP at baseline, and the use of antihypertensive drugs were regarded as covariates in a mixed models analysis. Results: CF ingestion did not affect any parameter. However, independent of the type of capsules ingested, a decrease in diastolic BP by 3 mmHg (95% CI: −4.0; −2.0) and an increase in the change in brachial artery diameter (pre vs. post occlusion) by 0.06 mm (95% CI: 0.01; 0.12) were detected in the non-diabetic group, while they remained unchanged in the T2DM group. Conclusion: No beneficial effects of CF were detected on vascular reactivity parameters in T2DM and non-diabetic participants.

## 1. Introduction

Type 2 diabetes mellitus (T2DM) and the cardiovascular morbidity and mortality associated with it represent an increasing worldwide health burden. The predicted rise in prevalence severely challenges economic and health care resources such that there is an equally increasing need for cheap and readily available interventions [1,2,3,4,5]. If effective, nutraceuticals, i.e., foods or compounds occurring in foods exerting medical or health benefits, might be worth considering for this purpose [6].

In the context of T2DM, flavonoids, and more specifically flavanols with its most abundant monomeric form epicatechin (EC), are of particular interest. These nutraceuticals found in cocoa, black tea, berries, and several other natural foods [7,8] are not only suggested to exert vasoprotective effects [9,10] but the physiological processes involved coincide with those implicated in the pathophysiology of T2DM related micro- and macrovasculopathies.

In T2DM, β-cell dysfunction and/or insulin resistance lead to chronic hyperglycemia [2,11]. This, in turn, increases oxidative stress, inflammation, and orthosympatic activity, and, more importantly, limits the availability of nitric oxide (NO), resulting in the above-mentioned micro- and macrovascular dysfunctions [12].

Flavanols derived from cocoa (CF) increase NO bioavailability and activity by enhancing endothelial NO synthase production and activity as well as by reducing oxidative stress through the inhibition of endothelial nicotinamide adenine dinucleotide phosphate oxidase [13,14]. Moreover, CF are suggested to inhibit endothelin-1, a strong vasoconstrictor [15], and to inhibit angiotensin-converting enzyme activity [16,17,18,19].

Indeed, in vitro, animal, and human research has suggested CF-induced amelioration of endothelial function [20,21,22] and blood pressure (BP) [23,24]. In addition, epidemiological studies have linked lower BP levels and decreased cardiovascular as well as all-cause mortality to cocoa consumption [25,26,27]. Moreover, several randomized control trials and meta-analyses implicated beneficial vascular properties of CF in different study populations [20,23,24,28,29,30]. This prompted the European Food Safety Authority to publish a Health Claim in 2012, stating that daily intake of 200 mg CF, concomitant with a normal, balanced diet, helps to protect the compliance of blood vessels, preserving a normal blood flow in the general population [31].

However, it is not clear to what extent this recommendation might be extrapolated to T2DM patients. In a recent meta-analysis, we found that evidence supporting the latter is weak at best: little, very heterogeneous research has produced inconsistent results [32]. We found weak evidence that mid/long-term CF ingestion decreased diastolic BP (DBP), but not systolic BP (SBP), by approximately 1–2 mmHg only. These effects were suggested to be greater in female, younger, and hypertensive adults, when EC content is at least 90 mg, and when CF is ingested in one daily batch. Additionally, the few trials examining acute CF effects are heterogeneous and inconsistent [33,34,35].

To distinguish whether the lack of results represents a lack of effectiveness of CF, or merely a lack of homogeneity in study methods and populations, we designed a basic protocol, using standardized measurements in a circumscript population using CF extracts in capsule form and placebo capsules in a cross-over study design.

We aimed to investigate whether the single ingestion of a high dose of CF induces an improvement in endothelial function (primary outcome), a reduction in BP, and/or an enhancement in muscle vasoreactivity in patients with T2DM compared to non-diabetic controls. This study also examined possible confounding effects of the use of antihypertensive drugs, specifically angiotensin-converting enzyme inhibitors (ACEi) and angiotensin receptor blockers (ARB), as these drugs are frequently used in T2DM subjects [36] and have intrinsic vasoactive effects.

We hypothesized that CF ingestion would improve micro- and macrovascular parameters in both non-diabetic and T2DM participants; however, stronger effects were expected in patients with T2DM and in participants taking antihypertensive drugs, as they suffer increased levels of oxidative stress and increased NO bioavailability.

## 2. Materials and Methods

As our research group recently published a protocol paper [37] describing the exact protocol of this acute, randomized, double-blinded, placebo-controlled cross-over study, only the main methodological parts are highlighted in this report.

This trial was approved by the ethical committee of Ghent University Hospital (April 2018) and was registered at clinicaltrials.gov (ID: NCT03722199). Written consent was obtained from all subjects before enrollment. Recruitment was conducted by spreading out flyers in Ghent and announcements on (social) media. Volunteers came from different parts in Flanders, Belgium. Data collection was performed from 5 October 2018 until 20 March 2021. However, since the COVID-19 pandemic forced the cancellation of all measurements for approximately 6 months and substantially hindered participation afterwards, data collection was strongly impeded in the last 12 months.

### 2.1. Participants

We considered two groups of participants (men and women, aged 18 to 85 years) for inclusion: (1) people with T2DM without micro- or macrovascular complications for at least 5 years (American Diabetes Association’s definition [38], with and without arterial hypertension, and (2) subjects without T2DM, with and without essential hypertension. Hypertensive subjects were only allowed to use ACEi or ARB, optionally combined with diuretics.

Exclusion criteria included active smoking or a history of smoking, either in the past 5 years or having more than 30 packyears; alcohol use (more than 10 units per week); chronic inflammatory diseases; active cancer; microvascular (retinopathy, nephropathy, or neuropathy) or macrovascular (cardiovascular, cerebrovascular, or peripheral artery diseases) diseases; neurological diseases; pregnancy; known cognitive impairment (e.g., dementia); language barrier (other than Dutch, French, or English); musculoskeletal disorders that could interfere with the outcomes of the measurements; and the use of NO-containing medication or phosphodiesterase type 5-inhibitors.

### 2.2. Trial Protocol

**Figure 1:**
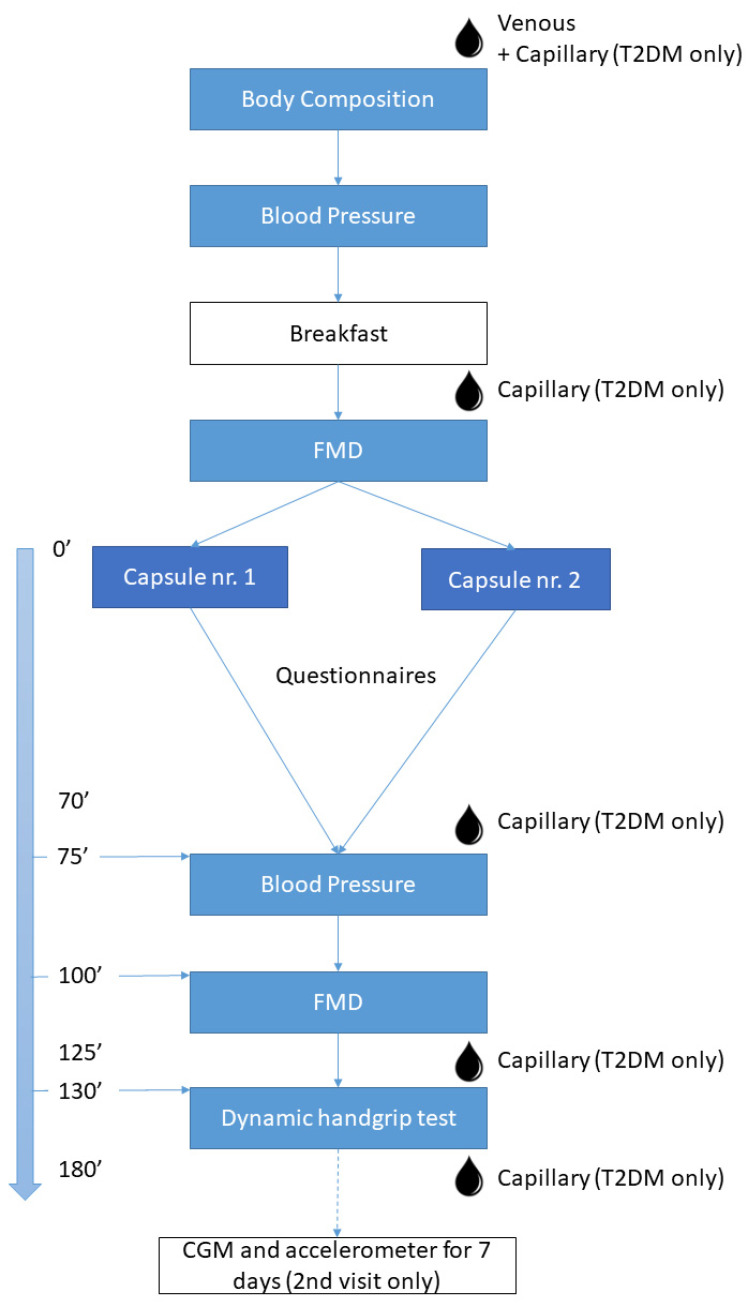
Flowchart. Legend: FMD = flow-mediated dilation test; CGM = continuous glucose monitoring system.

The study was designed as an acute randomized, double-blinded, placebo-controlled cross-over study. As presented in Figure 1, a standardized protocol was followed for 2 identical test days with the type of capsules as the only difference: CF-enriched capsules (2.5 g of cocoa extract containing 790 mg flavanols, 149 mg of which EC, Naturex SA, France) or placebo (maltodextrin and an equivalent dose of theobromine and caffeine compared to the CF-enriched capsules). The exact composition of each type of capsules was reported previously [37].

After an overnight fast (at least 8 h), blood samples (lipid profile, free fatty acids, hemogobinA1c (HbA1c), glucose, insulin, vitamin A, E, C, uric acid, C-reactive protein, and haptoglobin) were collected, and body composition was assessed. Immediately after, BP was recorded for 20 min, in 3 min intervals (Tango+, SunTech Medical, Morrisville, NC, USA), followed by a standardized breakfast (composition of meal: 18–21% protein, 20–37% fat, 44–59% carbohydrates, and 386–501 Kcal [37]; division of choices are listed in Table S1).

Thirty min after the start of the breakfast, a flow-mediated dilation (FMD)-test (GE, Vivid 7) was performed, after which either CF or placebo capsules were ingested (randomized order). After a 70-min resting period during which questionnaires (Autonomic Symptoms Profile Questionnaire, Epworth Sleepiness Scale) were filled out, a second BP measurement and FMD-test were executed. Subsequently, a dynamic handgrip exercise test (starting at 20% of maximal voluntary contraction and 10% increase each step, performed until exhaustion) with simultaneous monitoring of total hemoglobin (THb) variations via near-infrared spectroscopy (NIRS; OxiplexTS, ISS, Champaign, IL, USA) was completed.

In patients with T2DM, several capillary glycemic measurements were performed throughout the test day for safety reasons (no measurements during hypo- or clinically significant hyperglycemia (>250 mg/dL)).

Participants were asked to follow specific guidelines concerning physical activity and CF-low food and drink consumption starting from 3 days prior to each test day, as described previously [37].

Immediately following the second test day, participants wore an accelerometer (Actigraph wGT3X-BT, dominant side, hip (39)) and a Continuous Glucose Monitoring System (blind mode, IPro2, Medtronic, Northridge, CA, USA) for 7 consecutive days.

Study data was pseudonymized, collected, and managed using REDCap. Blood samples were stored in the medical biobank of the Ghent University Hospital (Bioresource center Ghent, Gent, Belgium, ID: BE 71067049) [39].

### 2.3. Statistical Analyses

Sample size calculations based on the reported results of Balzer et al. (2008) [33] were performed by SAS power and sample size. At least 9 subjects in each group were mandatory for 85% power based on the primary outcome.

To assess differences in baseline characteristics between both visits within and between each group (non-diabetic versus T2DM), paired *t*-tests or Wilcoxon signed-rank tests and independent *t*-tests or Mann–Whitney U tests were executed depending on the distribution of data. Both groups included patients with arterial hypertension using antihypertensive drugs (AHD). Therefore, we also further analyzed differences between and within groups based on the use of AHD. The same statistical tests were executed depending on the distribution of data; however, in the subgroups of the T2DM group, only non-parametric tests were used because of the small sample size.

Differences between non-diabetic and T2DM subjects concerning physical activity level by accelerometry and usual glycemic profile by continuous glucose monitoring were assessed via independent *t*-test or Mann–Whitney U tests, depending on the distribution of data.

For the analysis of CF effects on the macrovascular reactivity (FMD-test) and BP, a random intercept in mixed models was used with the following fixed effects: group (T2DM, non-diabetic); supplementation (CF-enriched, placebo); time of measurement during each visit (pre-, post-intake of capsules); as well as time × supplementation interaction, time × group interaction, and time × supplementation × group interaction. Differences between pre and post intake were calculated with a post hoc Sidak test if the model was significant or showed a tendency. Age, waist-to-hip ratio, and systolic BP (SBP) at baseline were added as covariates; however, SBP at baseline was not inserted for analyses of SBP, mean arterial pressure, and pulse pressure as they already include SBP. *p*-values and estimated coefficients, calculated by mixed models, were reported when a significant influence of covariates was detected.

For the analysis of CF effects on microvascular reactivity (dynamic handgrip exercise test results), a random intercept in mixed models was used as well, with the same covariates, but with other fixed effects because this test was only executed once at each visit: group (T2DM, non-diabetic); supplementation (CF-enriched, placebo); time of measurement during exercise test (increasing exercise steps with 10% of maximal voluntary contraction); as well as group × supplementation interaction, time × supplementation interaction, time × group interaction, and time × supplementation × group interaction.

Subsequently, a cofactor indicating the use of AHD was inserted to the models to analyze whether the use of AHD influences the observed effects.

In the case of dropouts or missing data, the participant was still included in data analysis providing one out of two visits was completed. The data of one complete visit was excluded because the participant (within the non-diabetic group) vomited after breakfast consumption in the CF condition. Similarly, data of two pre-CF FMD-measurements were excluded because of the unreliable tracing of diameter boundaries of the arteria brachialis (participants within the T2DM group).

The level of significance was set at *p* < 0.05 for all data analyses apart from the comparison of baseline characteristics between subgroups (based on the use of AHD) where the level of significance was set at *p* < 0.025. For all models with significant or almost significant (tendency) interactions, residuals were Gaussian. Data analyses were conducted using IBM SPSS statistics version 26(SPSS Inc., Chicago, IL, USA).

## 3. Results

### 3.1. Participants’ Characteristics

Eleven T2DM and twenty-four non-diabetic subjects were studied (Table 1). Of these individuals, 15 non-diabetic participants had hypertension treated with AHD, and 4 T2DM participants suffered from hypertension and used AHD. The baseline characteristics neither differed between T2DM and non-diabetic subjects nor between subgroups with or without AHD (Tables S2–S4), except for age, waist-to-hip ratio (WHR), and SBP, which were significantly higher in the 11 T2DM vs. 24 non-diabetic participants recruited (Table 1).

No statistical differences were detected for subjects’ characteristics between both visits.

Fasting blood results are listed in Table 2. HbA1c and fasting glucose were significantly higher in the T2DM group, while LDL- and total cholesterol were significantly higher in the non-diabetic group. Only minimal differences for fasting blood results between subgroups of T2DM subjects and non-diabetic T2DM subjects were detected (data of subgroups separately are presented in Supplementary Table S5).

Hyperglycemia (~200 mg/dL) was present in two T2DM persons (using AHD) just prior the first FMD measurement the day of placebo ingestion and in three T2DM persons just after the second FMD measurement (involving both visits with placebo or CF and participants with or without AHD use).

Physical activity levels were not significantly different between the two groups (Table 3). As expected, time in hyperglycemia (>180 mg/dL, >250 mg/dL), day-to-day glycemic excursions, and variability were significantly higher in the T2DM participants.

Physical activity data and results of the CGM were not significantly different in subgroups based on the use of AHD (within non-diabetic or T2DM) (data of four groups separately are presented in Supplementary Table S6).

### 3.2. Results of Primary and Secondary Measurements

#### 3.2.1. Macrovascular Reactivity: Flow-Mediated Dilation Test (FMD)—Primary Outcome 

**Figure 2:**
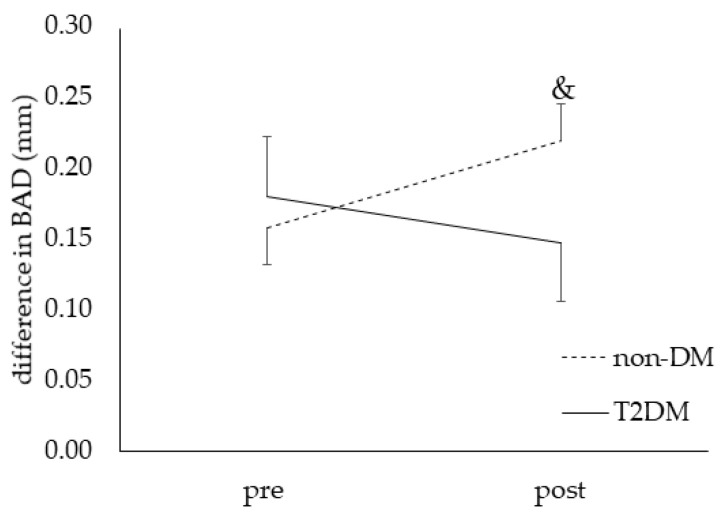
Difference in brachial artery diameter (BAD) in response to breakfast and capsules ingestion (cocoa flavanols and placebo combined) in both groups. Legend: Data are means ± SE. Main effects from mixed models when significant: time × group interaction for the difference in BAD (tendency, *p* = 0.07); post hoc analyses: no significant pairwise group differences, but a significant pairwise time difference in the non-diabetic group (*p* = 0.03; &); difference in BAD = BAD post cuff deflation minus BAD pre cuff inflation.

No significant effects of CF were detected on FMD outcome measures after correction for age, with WHR and SBP at baseline (Table 4). However, independent of capsule ingestion, the difference in BAD (peak BAD minus baseline BAD) showed a tendency towards a significant time × group interaction (*p* = 0.07). More in-depth analysis showed a significant increase from pre to post in the non-diabetic group, while this effect was not present in the T2DM group (61.7 µm; 95% CI: 6.5 −116.9 in non-diabetic vs. −32.5 µm; 95% CI: −118.9 −53.9 in T2DM group) (Figure 2). This tendency disappeared when the use of AHD was inserted as a cofactor.

#### 3.2.2. Blood Pressure Assessment—Secondary (Macrovascular) Outcome

**Figure 3:**
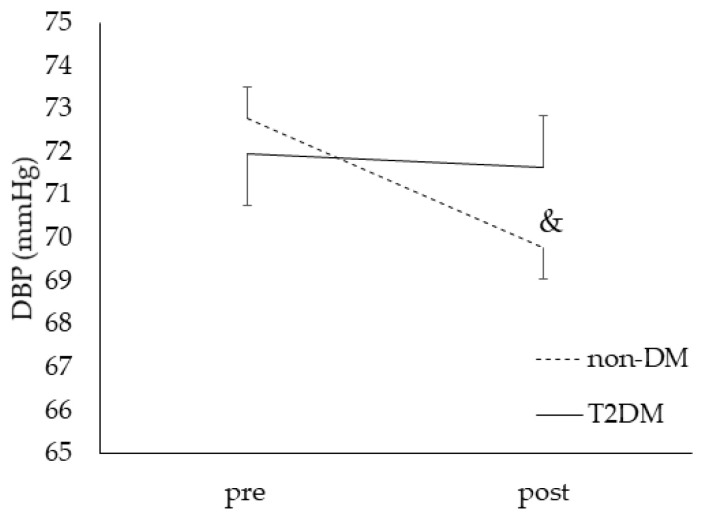
Diastolic blood pressure (DBP) in response to breakfast and capsules ingestion (cocoa flavanols and placebo combined in both groups). Legend: data are means ± SE. Main effects from mixed models when significant: time × group interaction, *p* = 0.01; post hoc analysis: no significant pairwise group differences, but a significant pairwise time difference in the non-diabetic group (*p* < 0.001; &).

No significant effect of CF was observed for SBP, DBP mean arterial pressure, pulse pressure or heart rate after correction for age, or WHR and/or SBP at baseline (only for HR) (Table 4). However, independent of capsule ingestion, a significant time × group interaction effect was seen for DBP (*p* = 0.01). Post hoc analysis showed a significant decrease of the DBP in the non-diabetic group after breakfast, which was not present in the diabetic group (−3 mmHg, 95% CI: −4.0; −2.0 in non-diabetic vs. −0.3 mmHg; 95% CI: −1.8; 1.2 in T2DM group) (Figure 3).

#### 3.2.3. Microvascular Reactivity: Dynamic Handgrip Strength Test with Near-Infrared Spectroscopy—Secondary Outcome 

**Figure 4:**
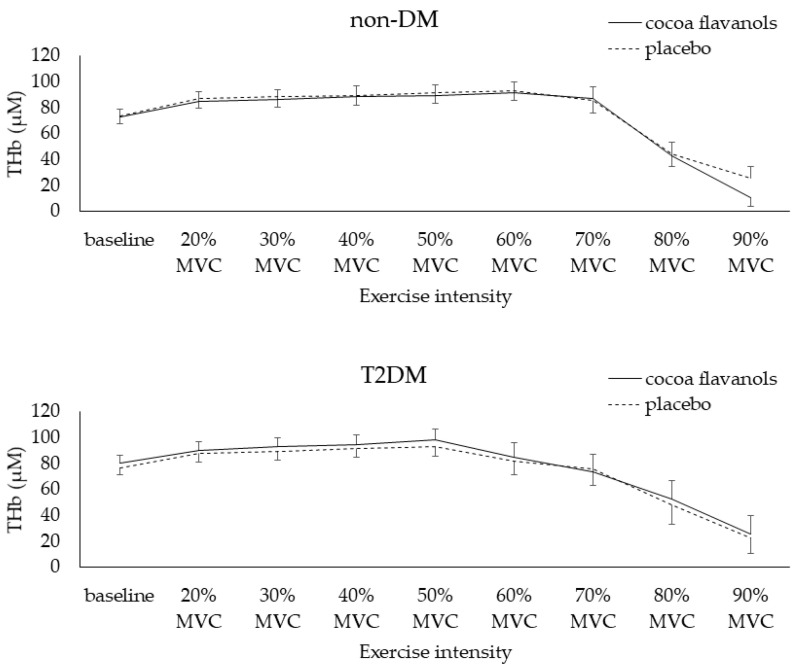
Muscle microvascular reactivity to the dynamic exercise test in both groups. Legend: data are means ± SE. MVC = maximal voluntary contraction; THb = total hemoglobin.

During the dynamic handgrip strength test, no significant interaction effect between exercise intensity, type of capsules ingested, and/or group was found for THb values, a factor reflecting the change in regional blood volume [41] (Table 5, Figure 4). However, when AHD use was added to the model, a significant AHD use × group × supplementation interaction was shown (*p* < 0.05). Post hoc analysis revealed significant higher THb levels during the entire exercise test in the non-diabetic individuals without the use of AHD after placebo ingestion compared to CF (*p* < 0.001). In the other groups, non-diabetic individuals with AHD and diabetic patients with or without AHD, no significant differences were shown.

## 4. Discussion

We aimed to assess whether the positive vascular effects of CF, as suggested in non-diabetic individuals, also occur in T2DM patients. In this acute, randomized, double-blinded, placebo-controlled cross-over study design, we found no additional effect of CF compared to placebo. The increase in BAD (+61.7 µm) and the small (−3 mmHg), but clinically relevant [42,43,44], decrease in DBP we measured occurred independent of CF and placebo capsules intake, and only in the non-diabetic group.

The absence of CF effects on vascular dynamic parameters might partly be explained by the small sample size. We based our power calculation on the study of Balzer et al. (2008) [33]; however, this study used a cocoa drink instead of purified CF capsules as the CF source, as well as a different placebo CF composition, which might have caused a biased calculation. Other possible reasons for a lack of effect include (1) the “acute” study design (one single CF ingestion compared to split doses throughout the day or ingestion for several days or weeks), (2) other methodological choices (e.g., post-prandial as opposed to fasting assessment), or (3) the choice of the CF source (purified CF capsules compared to chocolate beverages or bars). Furthermore, certain subject characteristics (such as age, fasting blood results, and drug use) might explain the divergent results compared to other studies. In our study, for matching reasons between groups, our non-diabetic participants were relatively older than in previous publications, had a higher weight with a mean BMI of 26.4 ± 4.5 kg·m^−2^, and the prevalence of insulin resistance was relatively high. This caused an overlap with the T2DM group, which might also partly account for a lack of difference between the T2DM and non-T2DM group.

The selection of T2DM individuals was specifically designed to be strict, especially concerning the absence of both macro- and microvascular conditions, in order to limit heterogeneity in our study population and to be able to address the presence of diabetes mellitus/chronic hyperglycemia as a pure, specific variable. This might, however, have reduced the detectability of possible CF effects, since these are reportedly more pronounced in persons with a certain level of vascular dysfunction [45,46,47]. Indeed, some have suggested that the effects of cocoa are only present in case SBP and DBP are above 140/80 mmHg. However, in our study, most participants had an SBP and DBP below this value. Perhaps in a population with vascular complications, CF effects would have been considerably more pronounced.

Nonetheless, we expected to find some vascular improvement from CF administration, if only in the non-diabetic group, and if only in FMD parameters, based on previous publications and especially considering the Health Claim of the European Food Safety Authority for the general population [31].

However, in this respect, it should be noted that the studies, upon which the above-mentioned Health Claim was based, were quite heterogenous. Not only did patient characteristics (e.g., sex, age, BMI, BP, smoking behavior) vary considerably but, in addition, there was a marked methodological heterogeneity in both interventional and control formulas: the former varied from beverages with milk or cold/hot water to chocolate bars and contained different amounts of CF as well as non-flavanol cocoa compounds. The latter varied from white chocolate, low CF dosed compounds, and compounds unmatched for non-flavanol components of cocoa to having no control at all.

This complicated comparability, not only amongst the studies, but also in relation to our study. For instance, when only looking at the analyzed acute trials, 9 out of 11 papers report on subjects between 25 and 55 years old [14,22,48,49,50,51,52,53,54]. In comparison, our population was aged 59.5 years (controls) and 66.7 years (T2DM group). So far, it is unclear to what extent this might explain the differences from our study: although age impacts vascular compliance [55], and therewith perhaps direct post-CF vascular reactivity, reports on age-dependent CF effects on vascular reactivity are contradictory. For instance, BP-reducing effects of cocoa products have been suggested to be greater in younger compared to older adults in some studies (CF effect in persons aged below compared to above 50 [23] and 65 years old [32]), but others have suggested the opposite [24]. However, these reports were based on long-term CF administration, and it is unclear to what extent these effects can be extrapolated to single CF ingestion.

For FMD, we found one study suggesting that single CF ingestion exerts greater acute (60–120 min) effects in older (>57.5 years) compared to younger adults (<57.5 years), whereas chronic (2–84 days) CF effects were not age-specific [20]. Our results showed no effect of age as a covariate.

As mentioned, other methodological challenges concerning the Health Claim involve the variation in intervention and placebo formulas in the papers analyzed. The varied use of chocolate introduces carbohydrates (e.g., sugar) and milk (whole milk or processed in chocolate), which may alter CF activity, effects on gastric emptying, and substance uptake, et cetera, and so complicate the comparability with our study but also between previous publications [23,56,57,58,59,60,61]. Moreover, several studies did not report on the exact amount of CF and/or did not control for non-flavanol components in cocoa, such as caffeine and theobromine [62,63,64].

Theobromine and caffeine are both methylxanthines with an intrinsic vascular impact, theobromine being a rather weak adenosine receptor antagonist [65,66]. It has been suggested that adding theobromine increases EC as well as CF effects on FMD [47] via its endothelium-independent vasodilating effects through cyclic nucleotide phosphodiesterases inhibition [67]. Our study matched CF and placebo capsules for theobromine and caffeine content, although the theobromine levels in our capsules (180 mg) might have been too low to exert synergistic effects with CF or EC, which is possibly seen by Balzer et al. (2008). They showed an increase of FMD through the single consumption of a cocoa drink containing comparable doses of CF and EC as in our study (371–963 mg, 78.9–203.0 mg, respectively) but higher doses of theobromine (575.6–586.2 mg) [68].

It is also worth mentioning that the Health Claim is solely dedicated to CF and not to other interfering components (e.g., caffeine and theobromine). Nevertheless, in our opinion, further research on synergistic, antagonistic, and/or supplementary actions with other cocoa components is required before drawing definite conclusions on the effects of cocoa ingestion on vascular health and on the contribution of CF, per se, hereon.

Furthermore, it should be noted that the Health Claim is based on papers performing a standardized FMD technique. Although it is recommended to measure FMD in a fasting state [69], this was not feasible in our study for practical reasons. It is possible that the consumed breakfast influenced our results. In the general healthy population, a decrease in FMD was demonstrated 1 h after glucose ingestion and was restored within 4 h [70]. This period of impaired endothelial function is in accordance with the postprandial rise in glucose levels, 1 to 3 h. Compared to healthy individuals, T2DM subjects have a delayed peak of insulin levels, which could possibly explain, in part, the observed differences between our non-diabetic vs. T2DM group [71]. As described, in the T2DM group, with and without the use of AHD, a few patients had a glycemia of around 200 mg/dL before an FMD test. In general, few researchers have examined postprandial CF effects; however, compared to everyday life, the postprandial state is the most physiological situation, as we live in this state most of the day [70].

As our study provided an intervention containing 790 mg CF, which is more than the recommended dose of 200 mg daily in the Health Claim, the impact of person characteristics as well as the impact of varied interventional and placebo formulas may not be ignored in cocoa research. Hence, we would plead for a nuanced interpretation of the current literature and the Heath Claim concerning the beneficial vascular effects of CF.

As already mentioned in the beginning of the discussion, a major limitation of the present study is the small sample size. Failure to complete groups as reported in our protocol paper [37] hinders the interpretation of the results. The recruitment of eligible participants was challenging by both the strict in- and exclusion criteria, but the COVID-19 pandemic hindered recruitment dramatically.

Considering the measurement of endothelial function (our primary outcome), FMD seems a proper measurement method, but this is a challenging technique and is highly operator dependent [69,72]. Training by a specialist in this technique was provided to ensure technical validation and good reproducibility. In addition, a software with automated tracking (together with optical control) was used for data analyses, as recommended. The intra- and inter-session coefficients of variation of the relative peak change in diameter (% FMD) were 23% and 32%, respectively, which is comparable with coefficients of variation described in other research [73]. Hence, a limiting factor of FMD tests is the rather broad noise and should be considered.

At this point, a practical guideline for dietary intake of cocoa cannot be formulated yet, although the European Food Safety Authority has published a Health Claim indicating daily doses of 200 mg CF. A non-linear dose-response relationship of cocoa on FMD has been established [20,74], but an ideal dose and the effectiveness in several patient populations remains a question. If vascular effects of CF are to be studied further, we believe that a correction is needed for all vasoactive components present in CF. We would also recommend using purified CF instead of chocolate formulas and report on the entire composition of interventional formulas with consumed foods or drinks. 

In conclusion, despite the paucity of effects of CF ingestion on peripheral micro- and macrovascular reactivity in our study, the congruent involvement of nitric oxide in both CF effects and the pathophysiology of vascular T2DM complications makes us reluctant to dismiss these cheap and easily accessible compounds as valuable nutraceuticals.

## Figures and Tables

**Table 1:** Participants’ characteristics.

	Non-DM	T2DM
N	24	11
Sex ( 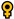 / 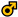 )	14/10	4/7
Age (years)	**59. 5 ± 5.5**	66.7 ± 6.1 *
Weight (kg)	77.1 ± 14.1	77.6 ± 13.7
BMI (kg·m^−2^)	26.4 ± 4.5	26.2 ± 3.0
Fat mass (%)	31.9 ± 9.1	29.7 ± 5.6
Waist-to-hip ratio	**0.91 ± 0.06**	0.95 ± 0.11 *
Baseline SBP (mmHg)	**116.0 ± 10.9**	125.1 ± 14.7 *
Baseline DBP (mmHg)	73.5 ± 7.5	75.1 ± 8.2
Baseline Mean arterial pressure (mmHg)	87.7 ± 8.4	91.8 ± 10.1
HbA1c (%)	5.8 ± 0.3	6.9 ± 0.7
Duration diabetes (years)	NA	9.3 ± 5.5
*Antihyperglycemic drugs (n):* Metformin Sulfonylurea DPP4- inhibitors GLP-1- RA SGLT-2 Insulin	NA	10 9 2 0 2 1 1
Duration of hypertension (years)	7.5 ± 5.5	9.0 ± 3.2
*Antihypertensive drugs (n)(%):* ACEi ARB	15 (62.5%) 11 4	4 (36.4%) 1 3
*Lipid-lowering drugs (n)(%):* HMG-CoA reductase inhibitors Fibrates	**5 (20.8%)** 4 1	7 (63.6%) * 7 0
History of smoking (years)	3.3 ± 6.7	8.8 ± 14.7 [0–40]

Data: means ± SD with [range] or frequencies at first visit; DPP4 = dipeptidylpeptidase-4; GLP-1-RA = glucagon-like peptide-1- receptor agonist; HMG-CoA = 3-hydroxy-3-methylglutaryl-coenzyme A; SGLT-2 = sodium glucose-cotransporter 2 inhibitors; * = *p*-value < 0.05 for difference between T2DM and non-diabetic.

**Table 2:** Fasting blood results.

	Non-DM		T2DM	
	CF	Placebo	CF	Placebo
Glucose (mg/dL)	**97.0 ± 8.0**	**96.2 ± 9.1**	127.7 ± 20.6 *	127.4 ± 17.0 *
Insulin (mU/L)	10.3 ± 6.6	9.8 ± 5.3	9.5 ± 6.1	9.5 ± 6.0
HOMA ^a^	2.4 ± 1.6	2.4 ± 1.4	3.1 ± 2.1	3.0 ± 1.8
Triglycerides (mg/dL)	109.9 ± 38.4	106.9 ± 42.1	**135.9 ± 72.2**	**112.1 ± 41.3** #
FFA (mmol/L)	0.55 ± 0.16	0.54 ± 0.20	0.57 ± 0.16	0.55 ± 0.15
HDL-cholesterol (mg/dL)	57.8 ± 18.7	57.9 ± 14.8	52.6 ± 14.4	53.8 ± 17.3
LDL-cholesterol (mg/dL) ^b^	**126.2 ± 35.8**	**124.2 ± 36.4**	89.7 ± 31.5 *	92.6 ± 30.7 *
Total cholesterol (mg/dL)	**206.0 ± 47.5**	**203.5 ± 45.9**	169.5 ± 39.5 *	168.8 ± 41.3 *
Uric acid (mg/dL)	4.7 ± 0.9	4.9 ± 1.3	5.1 ± 1.1	5.2 ± 1.0
CRP (mg/dL)	2.3 ± 3.4	2.8 ± 4.6	1.8 ± 1.0	1.5 ± 0.6
Vitamin C (mg/dL)	**0.7 ± 0.3**	0.7 ± 0.4	0.5 ± 0.3 *	0.6 ± 0.3
Vitamin A (µg/dL) ^c^ Vitamin E (mg/dL) ^c^	67.5 ± 13.1 1.3 ± 0.3		74.4 ± 15.2 1.2 ± 0.3	
Haptoglobin (g/L) ^c^	1.0 ± 0.4		1.2 ± 0.6	

Data means ± SD or frequencies; ^a^ calculated via fasting insulin x fasting glucose/22.5; ^b^ calculated via the Friedewald Formula; ^c^ only measured once, at first visit; CRP = C-reactive protein; FFA= free fatty acids; HDL = high density lipoprotein; LDL = low density lipoprotein. # = significant difference between both visits (CF versus placebo) within groups (*p* < 0.05); * = significant difference between T2DM and non-diabetic within condition (CF or placebo) (*p* < 0.05).

**Table 3:** Accelerometry and continuous glucose monitoring.

	Non-DM	T2DM
**Accelerometry:**		
Wearing time (min/day)	874.9 ± 16.5	842.2 ± 50.9
Valid days (days/week)	6.8 ± 0.5	6.6 ± 0.9
Step counts (n/day)	7521.1 ± 2690.3	6593.8 ± 2279.1
Moderate (min/week)	316.6 ± 219.5	186.4 ± 145.2
Vigorous (min/week)	6.3 ± 14.2	6.5 ± 17.4
Very vigorous (min/week)	0.8 ± 3.5	1.3 ± 4.2
MVPA (min/week)	323.7 ± 225.9	194.2 ± 149.3
**Continuous Glucose Monitoring System:**		
*Glycemic excursions:* % time in range (70–180 mg/dL) % time in hypoglycemic range <70 mg/dL % time in hyperglycemic range >180 mg/dL >250 mg/dL	**97.1 ± 3.4** **1.1 ± 1.8** **1.8 ± 3.0** **0.2 ± 0.4**	83.9 ± 14.0 * 0.7 ± 2.2 * 15.4 ± 14.1 * 1.4 ± 1.8 *
*Glycemic variability:* Coefficient of variation (%) ^a^ MAGE (mg/dL)	**17.4 ± 6.8** **45.1 ± 21.1**	22.9 ± 7.0 * 79.4 ± 23.7 *
Data sufficiency (%)	99.9 ± 0.4	99.9 ± 0.1

Data: means ± SD and [range]; ^a^ calculated by mean blood glucose divided by standard deviation; MAGE = mean amplitude of glycemic excursion; MVPA = moderate to vigorous physical activity; * = significant difference between groups (*p* < 0.05); some accelerometry data tended to be lower in the T2DM group compared with the non-diabetic group (*p* < 0.1 for wearing time, moderate activity, and MVPA).

**Table 4:** Results of examinations for macrovascular beds.

	Non-DM		T2DM	
	CF	Placebo	CF	Placebo
**Macrovascular reactivity:**				
**FMD-test: diameter of brachial artery (BAD)**				
*Baseline BAD (mm)*				
Before capsule Post capsule	3.8 ± 0.1 3.9 ± 0.1	3.8 ± 0.1 3.8 ± 0.1	3.9 ± 0.2 4.1 ± 0.2	4.1 ± 0.2 4.1 ± 0.3
*Peak BAD (mm)*				
Pre intake Post intake	4.0 ± 0.1 4.1 ± 0.2	3.9 ± 0.1 4.1 ± 0.1	4.2 ± 0.2 4.3 ± 0.2	4.2 ± 0.2 4.3 ± 0.2
*Difference BAD (µm) (peak–baseline)*				
Pre intake Post intake	**158.0 ± 25.7** **189.0 ± 32.9 &**	**161.3 ± 25.5** **244.4 ± 35.8 &**	260.5 ± 91.0 132.7 ± 29.7	165.6 ± 56.1 171.7 ± 30.4
*FMD (%) [((peak BAD–baseline BAD))/baseline BAD) x 100]*				
Pre intake Post intake	4.3 ± 0.8 4.8 ± 0.8	4.6 ± 0.9 6.7 ± 1.0	7.4 ± 2.9 3.3 ± 0.8	4.0 ± 1.2 4.6 ± 1.0
**Blood pressure (BP) and heart rate**				
*SBP (mmHg)* Pre intake Post intake	114.1 ± 2.6 110.4 ± 2.3	114.4 ± 2.4 110.3 ± 2.1	122.3 ± 3.7 119.6 ± 3.1	123.5 ± 4.5 121.8 ± 3.5
*DBP (mmHg)* Pre intake Post intake	**71.7 ± 1.7** **69.2 ± 1.6 &**	**72.4 ± 1.6** **68.8 ± 1.4 &**	73.4 ± 1.9 73.2 ± 1.6	74.0 ± 2.6 73.7 ± 1.8
*Mean arterial pressure [DBP + (1/3 x Pule pressure)]*				
Pre intake Post intake	85.8 ± 1.9 82.9 ± 1.7	86.4 ± 1.8 82.6 ± 1.6	89.7 ± 2.5 88.6 ± 2.0	90.5 ± 3.2 89.7 ± 2.3
*Pulse pressure (mmHg) (SBP–DBP)*				
Pre intake Post intake	42.4 ± 1.3 41.3 ± 1.1	41.9 ± 1.2 41.5 ± 1.1	48.9 ± 2.2 46.4 ± 1.9	49.4 ± 2.3 48.1 ± 2.2
*Heart rate (bpm)* Pre intake Post intake	62.1 ± 1.6 66.4 ± 1.7	64.5 ± 1.6 67.3 ± 1.7	65.0 ± 3.5 70.5 ± 3.6	63.6 ± 2.7 67.5 ± 2.9

Data are expressed as mean ± SE [range]; time × supplementation × group interactions, as well as time × supplementation interactions were not significant (whatever the cofactor use of AHD was included or not); a tendency (*p* = 0.07) for time × group interaction was detected for difference BAD and a significant (*p* = 0.01) time × group interaction appeared for DBP. Post hoc analyses: & = significant difference post vs. pre capsules ingestion, independent of the supplementation (i.e., the symbol & applies to CF and placebo combined).

**Table 5:** Results of examinations for microvascular beds.

	Non-DM				T2DM			
	Without AHD		With AHD		Without AHD		With AHD	
	CF	Placebo	CF	Placebo	CF	Placebo	CF	Placebo
**Microvascular reactivity:** **Muscle vasoreactivity to exercise**								
*THb (µM)* Baseline	63.1 ± 5.7	66.9 ± 7.8	77.4 ± 9.4	77.9 ± 9.3	81.3 ± 6.8	76.7 ± 6.2	79.2 ± 11.5	75.3 ± 9.9
Maximal	83.9 ± 7.7	90.9 ± 10.5	101.5 ± 12.4	102.3 ± 11.5	102.4 ± 8.7	93.6 ± 8.1	101.8 ± 16.3	100.7 ± 13.7
Difference (maximal–baseline)								
	**20.8 ± 2.5**	**24.0 ± 4.2 #**	24.1 ± 4.2	24.5 ± 3.1	21.1 ± 2.2	17.0 ± 2.5	22.6 ± 6.3	25.5 ± 3.9

Data are expressed as mean ± SE [range]; THb= total hemoglobin; only a significant (*p* < 0.001) group × supplementation × use of AHD interaction for THb; post hoc analyses: # = significant difference between type of capsules within subgroup (*p* < 0.05).

## Data Availability

Data are available on the request. Please contact the corresponding author.

## Supplementary Materials

The following supporting information can be downloaded at: https://www.mdpi.com/article/10.3390/nu14132692/s1, Table S1: Choice of breakfast-formulae; Table S2: Characteristics of participants title; Table S3: Autonomic Symptoms Profile Questionnaire; Table S4: Epworth Sleepiness Scale; Table S5: Fasting blood results; Table S6: Results of additional outcome measurements.
