# Supplementary material for: Acute Effects of Cocoa Flavanols on Blood Pressure and Peripheral Vascular Reactivity in Type 2 Diabetes Mellitus and Essential Hypertension
Source: Nutrients. 2022 Jun 28;14(13):2692. doi: 10.3390/nu14132692 (PMC9268419; doi:10.3390/nu14132692)
Supplement: Supplementary file 1 [file nutrients-14-02692-s001.zip › nutrients-1776187-supplementary.pdf]

Supplementary Material

Table S1: Choice of breakfast-formulae

	Non-DM		T2DM	
	**Without AHD**	**With AHD**	**Without AHD**	**With AHD**
Formula 1: cereals	1	1	0	0
Formula 2: yoghurt	4	4	3	1
Formula 3: milk	1	4	3	1
Formula 4: gingerbread	3	6	1	2

Data: frequencies; AHD= antihypertensive drugs; formula 1= 60 g cereals (Special K, Kellogg's), 200 g semi-skimmed milk, 125 g semi-skimmed cottage cheese; formula 2= 90 g light brown bread, 15 g butter (Halvarine, Blue Band), 17 g cream cheese (La vache qui rit), 15 g jam (reduced sugars), 125 g low-fat yoghurt; formula 3= 90 g light brown bread, 200 g semi-skimmed milk, 15 g butter (Halvarine, Blue Band), 15 g jam (reduced sugars), 30 g cheese (Gouda, Hollandic); formula 4= 90 g light brown bread, 15 g butter (Halvarine, Blue Band), 25 g gingerbread (reduced sugars), 30 g cheese (Gouda, Hollandic), 125 g low-fat yoghurt. For nutrient composition we refer to our published protocol-paper [1].

Table S2: Characteristics of participants

	Non-DM		T2DM	
	**Without AHD**	**With AHD**	**Without AHD**	**With AHD**
N	9	15	7	4
Sex (♀/♂)	5/4	9/6	3/4	1/3
Age (years)	59.7 ± 5.3	59.3 ± 5.8	67.7 ± 5.9	65.0 ± 7.0
Weight (kg)	75.8 ± 13.1	77.8 ± 15.0	74.6 ± 15.8	82.9 ± 8.1
BMI (kg/m^2) [range]	25.7 ± 3.9 [20.9 – 32.7]	26.8 ± 5.0 [18.2 – 35.5]	25.5 ± 3.5 [21.1 – 31.9]	27.5 ± 1.8 [24.9 – 29.0]
Fatmass (%)	31.6 ± 9.7	32.1 ± 9.1	29.7 ± 5.8	29.6 ± 6.0
Waist-to-hip ratio	0.90 ± 0.06	0.91 ± 0.07	0.92 ± 0.14	0.98 ± 0.02
Baseline SBP (mmHg) [range]	115.4 ± 10.7 [99.0 – 132.3]	116.4 ± 11.3 [100.2 – 141.5]	121.2 ± 17.0 [91.2 – 143.7]	131.9 ± 6.7 [125.3 – 137.8]
Baseline DBP (mmHg) [range]	72,0 ± 6,7 [61.3 – 85.3]	74.4 ± 8.0 [60.3 – 89.0]	73.7 ± 9.4 [58.8 – 88.0]	77.6 ± 5.8 [72.7 – 84.5]
Baseline mean arterial pressure (mmHg) [range]	86.5 ± 7.8 [74.3 – 101.0]	88.4 ± 8.9 [73.7 – 103.4]	89.5 ± 11.8 [69.6 – 106.6]	95.7 ± 5.0 [90.3 – 102.3]
HbA1c (%)	5.7 ± 0.4	5.9 ± 0.2	7.1 ± 0.7	6.7 ± 0.5
Duration diabetes (years) [range]	NA	NA	9.3 ± 6.4 [5 – 23]	9.3 ± 4.3 [6 – 15]
Antihyperglycemic drugs (n)	NA	NA	**6**	**4**
-Metformin			5	4
-Sulfonylurea			1	1
-DPP4-inhibitors			0	0
-GLP-1-RA			1	1
-SGLT-2			1	0
-Insulin			1	0
Duration of hypertension (years)[range]	NA	7.5 ± 5.5 [2 – 20]	NA	9.0 ± 3.2 [6 – 13]
Antihypertensive drugs (n)(%)	NA	**15** (62.5%)	NA	**4** (36.4%)
-ACEi		11		1
-ARB		4		3
Lipid-lowering drugs (n)(%)	**2**	**3**	**4**	**3**
-HMG-CoA reductase inhibitors	1	3	4	3
-Fibrates	1	0	0	0
History of smoking (n)	2.2 ± 5.1	3.9 ± 7.6	10.2 ± 16.6	6.3 ± 12.5
Autonomic profile[a]				
-Orthostatic intolerance	2.7 ± 5.3	1.9 ± 5.0	1.7 ± 4.5	4.0 ± 8.0
-Vasomotor function	0.0 ± 0.0	0.3 ± 0.9	0.1 ± 0.3	0.6 ± 1.2
Daytime sleepiness[b]	5.9 ± 3.0	8.2 ± 3.8	6.3 ± 2.4	4.8 ± 1.0

Data: means ± SD or frequencies at first visit; [a]data obtained through the Autonomic Symptoms Profile Questionnaire; [b]data obtained through the Epworth Sleepiness Scale; DPP4= dipeptidylpeptidase-4; GLP-1=glucagon-like peptide-1-receptor agonist; HMG-CoA= 3-hydroxy-3-methylglutaryl-coenzyme A; SGLT-2= sodium glucose-cotransporter 2 inhibitors. No differences between subgroups of T2DM subjects, neither between subgroups of non-diabetic subjects.

Table S3: Autonomic Symptoms Profile Questionnaire

ID	Ortostatic	Vaso-motor	Secreto-motor	Gastro-intestinal	Bladder	Pupillo-motor	Total
**Non-DM without AHD**							
ngnb101	0	0	6.42	10.68	0	1.65	18.75
ngnb103	0	0	2.14	0.89	0	2.64	5.67
ngnb204	0	0	6.42	0	0	0	6.42
ngnb205	0	0	2.14	0	0	0.33	2.47
ngnb206	0	0	2.14	0.89	1.11	0.66	4.8
ngnb207	0	0	0	2.67	3.33	1.65	7.65
ngnb108	12	0	0	5.34	1.11	0	18.45
ngnb109	0	0	6.42	1.78	0	1.98	10.18
ngnb111	12	0	0	2.67	0	0.99	15.66
**Non-DM with AHD**							
ngac201	12	0	0	0.89	0	1.32	14.21
ngac102	0	0	2.14	0	1.11	1.65	4.9
ngac104	0	0	0	4.45	0	0	4.45
ngac105	16	1.66	2.14	0.89	2.22	2.64	25.55
ngac206	0	0	0	3.56	1.11	1.65	6.32
ngac107	0	0	2.14	0.89	1.11	0	4.14
ngac108	0	0	2.14	0	1.11	2.64	5.89
ngac109	0	0	0	3.56	0	1.32	4.88
ngac110	0	0	2.14	0	0	0.99	3.13
ngac211	0	0	0	0.89	1.11	1.32	3.32
ngac212	0	0	0	3.56	0	0	3.56
ngab101	0	3.32	8.56	0.89	2.22	2.97	17.96
ngab202	0	0	2.14	1.78	0	0	3.92
ngab203	0	0	2.14	1.78	2.22	0.99	7.13
ngab104	0	0	2.14	0.89	0	0.66	3.69
**T2DM without AHD**							
dmac201	0	0	8.56	3.56	3.33	0	15.45
dmnb101	0	0	4.28	1.78	0	1.65	7.71
dmnb103	12	0.83	4.28	2.67	3.33	1.32	24.43
dmnb204	0	0	0	2.67	0	0	2.67
dmnb105	0	0	8.56	8.01	0	2.31	18.88
ngnb202	0	0	2.14	0	1.11	0.99	4.24
dmnb207	0	0	2.14	3.56	0	0.99	6.69
**T2DM with AHD**							
dmab201	16	0	2.14	2.67	4.44	1.98	27.23
dmab202	0	0	0	5.34	0	1.32	6.66
dmab103	0	2.49	6.42	4.45	1.11	1.32	15.79
dmac202	0	0	0	0.89	0	0	0.89

Scoring of questionnaire was done via Sletten et al. (2012) [2], each column represents the total score of the participants within each domain and the last column represents the sum of the scores of all domains multiplied with their Cronbach α coefficient; AHD= antihypertensive drugs.

Table S4: Epworth Sleepiness Scale

ID	ESS_1	ESS_2	ESS_3	ESS_4	ESS_5	ESS_6	ESS_7	ESS_8	ESS_total
**Non-DM without AHD**									
ngnb101	1	3	1	1	2	0	1	0	9
ngnb103	0	3	1	2	2	0	1	0	9
ngnb204	0	0	1	0	1	0	0	0	2
ngnb205	1	2	1	1	2	0	1	0	8
ngnb206	0	1	0	1	1	0	0	0	3
ngnb207	2	3	1	0	2	0	1	0	9
ngnb108	1	1	0	0	3	0	0	0	5
ngnb109	0	1	0	0	1	0	0	0	2
ngnb111	2	3	0	0	1	0	0	0	6
**Non-DM with AHD**									
ngac201	2	0	0	0	2	0	0	0	4
ngac102	2	2	2	2	2	1	2	2	15
ngac104	3	3	0	1	3	0	0	0	10
ngac105	2	2	0	0	2	0	0	0	6
ngac206	1	0	0	3	3	0	2	0	9
ngac107	0	3	1	0	2	0	3	0	9
ngac108	3	3	2	2	2	1	2	2	17
ngac109	1	1	0	0	2	0	1	0	5
ngac110	1	2	0	1	3	0	0	0	7
ngac211	1	2	0	missing	3	0	2	0	8
ngac212	0	2	0	0	3	0	0	0	5
ngab101	missing	missing	missing	missing	missing	missing	missing	missing	missing
ngab202	1	3	0	1	2	0	1	0	8
ngab203	1	2	0	0	2	0	2	0	7
ngab104	1	0	0	0	2	0	2	0	5
**T2DM without AHD**									
dmac201	3	2	1	1	2	0	0	0	9
dmnb101	1	3	1	missing	3	0	0	0	8
dmnb103	0	0	0	0	3	0	0	0	3
dmnb204	0	2	1	0	2	0	1	0	6
dmnb105	0	3	0	0	3	0	1	0	7
ngnb202	0	1	0	1	1	0	0	0	3
dmnb207	2	3	0	0	3	0	0	0	8
**T2DM with AHD**									
dmab201	0	2	0	0	2	0	0	0	4
dmab202	missing	2	0	1	2	0	1	0	6
dmab103	1	2	0	0	1	0	0	0	4
dmac202	0	2	0	0	2	0	1	0	5

Scoring of questionnaire was done via Sander et al. (2016) [3]; *AHD= antihypertensive drugs; ESS_x= represents number of question within the questionnaire 'Epworth Sleepiness Scale'; ESS_total= represents total score on the questionnaire, calculated via sum of each ESS_x.*

Table S5: Fasting blood results

	Non-DM				T2DM			
	Without AHD		With AHD		Without AHD		With AHD	
	CF	placebo	CF	placebo	CF	placebo	CF	placebo
Glucose (fasting) (mg/dL)	**94.2 ± 9.1**	**90.0 ± 8.0 ∫**	98.8 ± 6.8	**100.5 ± 7.4***	125.9 ± 19.0	124.6 ± 10.9	131.0 ± 25.7	132.3 ± 26.0
Insulin (mU/L)	9.0 ± 7.7	7.9 ± 5.3	11.1 ± 5.8	11.1 ± 5.2	8.0 ± 7.1	7.6 ± 5.3	12.2 ± 2.7	12.9 ± 6.1
HOMA[a]	2.2 ± 2.0	1.8 ± 1.3	2.6 ± 1.4	2.8 ± 1.3	2.6 ± 2.5	2.4 ± 1.7	3.9 ± 1.0	4.1 ± 1.6
QUICKI[b]	0.35 ± 0.03	0.36 ± 0.03	0.34 ± 0.02	0.33 ± 0.02	0.35 ± 0.05	0.35 ± 0.04	0.31 ± 0.01	0.31 ± 0.02
Uric acid (mg/dL)	4.5 ± 0.9	4.6 ± 1.0	4.8 ± 1.0	5.0 ± 1.4	4.9 ± 1.3	5.0 ± 1.2	5.3 ± 0.7	5.5 ± 0.7
Triglycerides (mg/dL)	107.3 ± 36.4	92.6 ± 24.3	111.4 ± 40.7	116.1 ± 49.0	115.4 ± 48.7	104,4 ± 36.0	171.6 ± 99.8	125.6 ± 52.1
FFA (nmol/L)	0.57 ± 0.12	0.48 ± 0.10	0.54 ± 0.19	0.59 ± 0.23	0.66 ± 0.12	**0.63 ± 0.11**	0.43 ± 0.11	**0.42 ± 0.08***
HDL-cholesterol (mg/dL)	56.7 ± 12.6	56.1 ± 12.8	58.5 ± 22.0	59.1 ± 16.2	51.2 ± 15.7	52.5 ± 19.2	55.1 ± 13.6	56.1 ± 15.9
LDL-cholesterol (mg/dL)[c]	121.2 ± 30.1	121.9 ± 28.7	129.2 ± 39.6	125.7 ± 41.6	96.5 ± 36.8	97.6 ± 38.1	78.0 ± 17.7	83.8 ± 8.5
Total cholesterol (mg/dL)	199.4 ± 38.0	196.6 ± 36.9	210.0 ± 53.2	208.0 ± 51.7	170.7 ± 48.2	171.0 ± 50.6	167.4 ± 23.3	165.0 ± 23.4
CRP (mg/dL)	**1.1 ± 0.6**	2.2 ± 2.9	**3.0 ± 4.1***	3.1 ± 5.5	1.6 ± 0.6	1.7 ± 0.6	2.1 ± 1.6	1.2 ± 0.5
Vitamin C (mg/dL)	0.6 ± 0.3	0.7 ± 0.4	0.7 ± 0.3	0.7 ± 0.3	0.7 ± 0.3	0.7 ± 0.3	0.4 ± 0.1	0.4 ± 0.2
Vitamin A (µg/dL)[d]	61.8 ± 9.7		70.9 ± 14.0		74.5 ± 16.6		74.2 ± 14.8	
Vitamin E (mg/dL)[d]	1.2 ± 0.2		1.3 ± 0.4		1.2 ± 0.3		1.2 ± 0.3	
Haptoglobin (g/L)[d]	0.8 ± 0.3		1.1 ± 0.4		1.2 ± 0.7		1.2 ± 0.5	

*Data: means ± standard deviation (SD) or frequencies at first visit unless specified differently; [a]calculated via fasting insulin (mU/L) x fasting glucose (mg/dL) / 405; [b]calculated via 1/(log(insulin (mU/L))+log(glucose (mg/dL))); [c]calculated via the Friedewald Formula; [d]only measured once, at first visit; AHD= antihypertensive drugs; CF= cocoa flavanols; CRP= C-reactive protein; FFA= free fatty acids; HDL= high density lipoprotein; LDL= low density lipoprotein; *= differences between subgroups (p<0.025); ∫= significant difference between both visits (p<0.025).*

Table S6: Results of additional outcome measurements

	Non-DM		T2DM	
	Without AHD	With AHD	Without AHD	With AHD
**Accelerometry:**				
Wearing time (minutes/day)	867.5 ± 21.4	879.4 ± 11.3	826.6 ± 64.8	869.5 ± 25.3
Valid days (days/week)	6.9 ± 0.3	6.8 ± 0.6	6.9 ± 0.4	6.2 ± 1.5
Step counts (n/day)	7677.7 ± 2041.1	7427.1 ± 3079.7	6593.9 ± 2239.0	6593.6 ± 2699.6
Moderate (minutes/week)	356.2 ± 178.5	292.8 ± 243.6	194.9 ± 136.0	171.5 ± 181.3
Vigorous (minutes/week)	6.7 ± 14.3	6.1 ± 14.7	8.6 ± 21.8	3.0 ± 5.4
Very vigorous (minutes/week)	0.1 ± 0.3	1.1 ± 4.4	0.0 ± 0.0	3.5 ± 7.0
MVPA (minutes/week)	363.0 ± 181.5	300.1 ± 251.9	203.4 ± 140.8	178.0 ± 184.6
**Continuous Glucose Monitoring System:**				
Glycemic excursions:				
% time in range (70-180 mg/dL)	96.3 ± 4.6	97.6 ± 2.6	89.7 ± 10.6	70.5 ± 12.8
% time in hypoglycemic range				
<70 mg/dL	1.4 ± 2.2	1.0 ± 1.5	0.0 ± 0.0	2.3 ± 4.1
% time in hyperglycemic range				
>180 mg/dL	2.3 ± 4.1	1.4 ± 2.2	10.3 ± 10.6	27.2 ± 16.2
>250 mg/dL	0.2 ± 0.6	0.1 ± 0.2	0.9 ± 1.2	2.5 ± 2.6
Area under curve (mg/dL/minute)				
Below 70mg/dL	0.1 ± 0.2	0.1 ± 0.1	0.0 ± 0.0	0.3 ± 0.6
Above 180 mg/dL	0.7 ± 1.2	0.3 ± 0.6	3.0 ± 3.4	8.4 ± 5.5
Above 250 mg/dL	0.1 ± 0.3	0.0 ± 0.1	0.2 ± 0.4	1.2 ± 1.1
Glycemic variability:				
Standard deviation (mg/dL)	20.5 ± 10.0	19.3 ± 7.3	29.4 ± 8.9	40.3 ± 8.7
Coefficient of variation (%)[a]	18.1 ± 8.1	16.9 ± 6.1	21.1 ± 5.4	27.0 ± 9.8
MAGE (mg/dL)	46.8 ± 23.9	44.0 ± 20.1	71.8 ± 22.3	97.0 ± 19.6
Low blood glucose index	0.7 ± 0.6	0.5 ± 0.4	0.1 ± 0.1	0.6 ± 1.0
High blood glucose index	0.7 ± 0.9	0.6 ± 0.5	2.7 ± 1.6	5.4 ± 2.8
Data sufficiency (%)	100.0 ± 0.0	99.8 ± 0.5	99.9 ± 0.1	99.8 ± 0.2
Days CGM worn (n)	6.0 ± 0.0	5.8 ± 0.6	6.0 ± 0.0	6.0 ± 0.0

Data: means ± standard deviation (minimum – maximum); [a]calculated by mean blood glucose divided by standard deviation; AHD= antihypertensive drugs; MAGE= mean amplitude of glycemic excursions; MVPA= moderate to vigorous physical activity. No significant differences between subgroups of T2DM subjects, neither between subgroups of non-diabetic subjects.

References

1. Tanghe A, Celie B, Shadid S, Rietzschel E, Op't Roodt J, Reesink KD, et al. Acute Effects of Cocoa Flavanols on Blood Pressure and Peripheral Vascular Reactivity in Type 2 Diabetes Mellitus and Essential Hypertension: A Protocol for an Acute, Randomized, Double-Blinded, Placebo-Controlled Cross-Over Trial. *Frontiers in cardiovascular medicine* (2021) 8:152.

2. Sletten DM, Suarez GA, Low PA, Mandrekar J, Singer W, editors. COMPASS 31: a refined and abbreviated Composite Autonomic Symptom Score. *Mayo Clinic Proceedings*; 2012: Elsevier.

3. Sander C, Hegerl U, Wirkner K, Walter N, Kocalevent R-D, Petrowski K, et al. Normative values of the Epworth Sleepiness Scale (ESS), derived from a large German sample. *Sleep and Breathing* (2016) 20(4):1337-45.
